# Ocular Manifestations of Emerging Flaviviruses and the Blood-Retinal Barrier

**DOI:** 10.3390/v10100530

**Published:** 2018-09-28

**Authors:** Sneha Singh, Ashok Kumar

**Affiliations:** 1 Department of Ophthalmology, Visual and Anatomical Sciences, Wayne State University, Detroit, MI 48201, USA; gq8860@wayne.edu; 2 Department of Biochemistry, Microbiology, and Immunology, Wayne State University, Detroit, MI 48201, USA

**Keywords:** flavivirus, eye, zika virus, blood-retinal barrier, ocular, innate response

## Abstract

Despite flaviviruses remaining the leading cause of systemic human infections worldwide, ocular manifestations of these mosquito-transmitted viruses are considered relatively uncommon in part due to under-reporting. However, recent outbreaks of Zika virus (ZIKV) implicated in causing multiple ocular abnormalities, such as conjunctivitis, retinal hemorrhages, chorioretinal atrophy, posterior uveitis, optic neuritis, and maculopathies, has rejuvenated a significant interest in understanding the pathogenesis of flaviviruses, including ZIKV, in the eye. In this review, first, we summarize the current knowledge of the major flaviviruses (Dengue, West Nile, Yellow Fever, and Japanese Encephalitis) reported to cause ocular manifestations in humans with emphasis on recent ZIKV outbreaks. Second, being an immune privilege organ, the eye is protected from systemic infections by the presence of blood-retinal barriers (BRB). Hence, we discuss how flaviviruses modulate retinal innate response and breach the protective BRB to cause ocular or retinal pathology. Finally, we describe recently identified infection signatures of ZIKV and discuss whether these system biology-predicted genes or signaling pathways (e.g., cellular metabolism) could contribute to the pathogenesis of ocular manifestations and assist in the development of ocular antiviral therapies against ZIKV and other flaviviruses.

## 1. Introduction

Flaviviruses consist of more than 90 RNA-enveloped viruses out of which 30 can cause severe disease in humans and animals. Most members of the family are arthropod-borne and are transmitted to the host by mosquitos or ticks [1]. The genus *flavivirus* contains several members which continue to be of global concern such as mosquito-borne Dengue virus (DENV 1–4), West Nile Virus (WNV), Japanese Encephalitis virus (JEV), Yellow fever virus (YFV), Zika virus (ZIKV), Murray Valley Encephalitis Virus (MVEV), Kyasanur Forest Disease virus (KFDV), St. Louis Encephalitis Virus (SLEV) (Fenner’s Veterinary Virology, 5th ed, 2017) along with Tick-borne Encephalitis Virus (TBEV). Interestingly, despite sharing a similar genomic organization and replication mechanisms, phylogenetically closely related flaviviruses can induce a spectrum of diseases. Broadly, the genus *flavivirus* is known to cause hemorrhage and vascular leakage (e.g., DENV and YFV), encephalitis (e.g., WNV and JEV) and, more recently, known to cause microcephaly and Guillain-Barre syndrome (ZIKV) [2,3].

**Figure 1:**
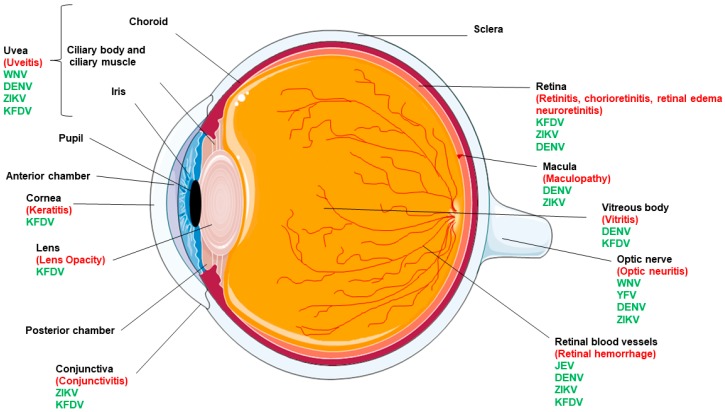
Eye anatomy and ocular complications caused by flaviviruses. Various components of the human eye are labelled in black. The flaviviruses responsible for causing ocular manifestations are shown in green whereas specific ocular tissue pathology is highlighted in red.

Apart from causing systemic infections, these pathogens have been documented to cause multiple ocular complications (Figure 1) with the most common being conjunctivitis, uveitis, and diseases in the posterior segment of the eye (e.g., choroiditis, chorioretinal atrophy, retinitis) [4] (Table 1).

In the following sections, we provide an overview of ocular complications resulting from flavivirus infections in humans worldwide and elaborate on their main features, systemic complications, and ocular involvement. These viruses have evolved specific mechanisms to counteract the antiviral response from the host and exploit various metabolic pathways to cause disease. Moreover, we discuss probable interactions of viruses and their encoded proteins with ocular cells and the BRB to infect the eye and cause ophthalmic anomalies.

### Molecular Pathogenesis of Flaviviruses

Flaviviruses are a family of lipid-enveloped viruses with a single-stranded ~10.5 kb positive-sense RNA genome. They encode only ten proteins which exploit host machinery to complete their infectious replication cycles. Most viral proteins have been shown to associate with host cellular functions and metabolic pathways but their biological consequence in most of these communications are not yet clearly understood [2]. The virus is introduced into the host by an infected vector (e.g., mosquito) during its blood meal. Flaviruses enter cells by receptor-mediated endocytosis [2], where they bind with host endosomes in an acidic environment triggering conformational modifications to their envelope (E) glycoprotein. Conformational changes lead to fusion of the host and viral membrane, facilitating the release of the viral genomic RNA. The polypeptide is co- and post-translationally processed by host signalases and virus encoded serine proteases to translate into the ten viral proteins: three structural proteins (Envelope (E), Capsid (C), Pre-Membrane (PrM)) and seven non-structural proteins (NS1, NS2A, NS2B, NS3, NS4A, NS4B and NS5) [1].

**Figure 2:**
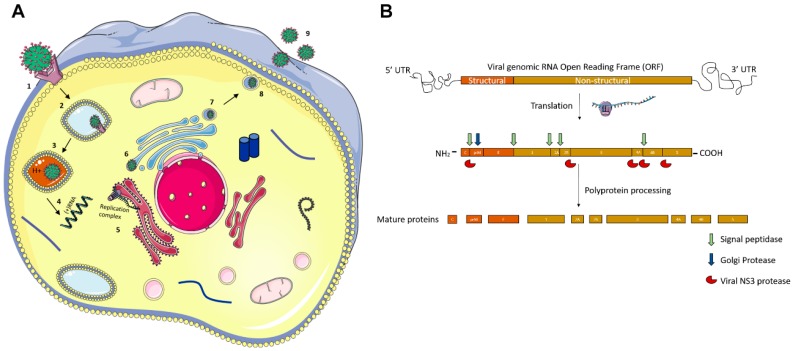
Flavivirus replication cycle and genome structure. (**A**) The flavivirus enters the host cell by attaching to specific receptors (1) which then leads to its endocytosis (2) followed by fusion to a lysosome into an acidic environment (3). The genome is released from the endolysosome (4) which is then translated on the Endoplasmic reticulum membrane (5) and post translational processing is done in the Golgi apparatus (6). The mature virus then buds off from the Golgi network (7) to the extracellular space via exocytosis (8, 9). (**B**) The genome consists of three structural proteins (Envelope (E), Capsid (C) and pre-membrane (prM)) and seven non-structural proteins (NS1, NS2A, NS2B, NS3, NS4A, NSB, and NS5).

The non-structural proteins are involved in viral genome replication, budding, and deploying the host cell machinery. Following translation, the RNA-dependent RNA polymerase (RdRp), NS5, creates a negative-strand from genomic RNA, which then becomes a template for a new positive strand to be made. In the rough Endoplasmic Reticulum (ER), viral proteins begin to assemble, and viral RNA is packaged with structural proteins—C, E, and prM. Viral particles are then transported to the trans-Golgi network, where prM is cleaved into M, and the mature virus is then released from the host via exocytosis into the extracellular space (Figure 2).

## 2. Flaviviruses and Ocular Complications

Despite the plethora of studies on host–virus interactions to understand their pathogenesis in humans, studies of flaviviruses and their role in ocular diseases are limited. Our knowledge is incomplete regarding properties which confer an ocular tropism to particular flaviviruses and underlying molecular mechanisms which allow the breach of blood-retinal barrier (BRB) for ocular exposure. To appropriately control and treat diseases presenting with ocular complications, a more rigorous understanding of ocular symptoms is needed that can range from maculopathy to retinal hemorrhage and vision loss (Table 2).

### 2.1. Yellow Fever Virus (YFV)

Yellow fever (YF) has been a major threat to human health from the 18th to 20th century with repeated epidemics in North America, Europe, and the Caribbean. Described as “the original viral hemorrhagic fever (VHF),” severe YF is pan-systemic viral sepsis with viremia, fever, prostration, hepatic, renal and myocardial injury, hemorrhage, shock and lethality up to 20–50% [44]. There is an abrupt onset of severe symptoms after 3 to 6 days of fever. Patients may experience fever, chills, malaise, headache, lower back pain, generalized myalgia, nausea, and dizziness, often manifesting Faget’s sign (increasing temperature with decreasing pulse rate). Infections in humans range from unapparent abortive infection to a fatal, fulminating disease with high fever, vomiting, epigastric pain, prostration, and dehydration. Hepatic-induced coagulopathy produces severe hemorrhagic manifestations including petechiae, ecchymosis, epistaxis (bleeding of the gums), and the characteristic “black vomit” (hematemesis; gastrointestinal hemorrhage) [10]. The infection rate of YFV has rapidly declined due to the successful development of two attenuated vaccines during the 1930–1940s [9]. Currently, YFV is majorly persevered in jungle environments with sporadic human outbreaks in South America and sub-Saharan Africa. The present risk of emergence and transmission of the disease is being primarily controlled by wide coverage of vulnerable populations with vaccinations [45,46].

Among reported studies in virus-induced ocular complications, there is only a single case study on a 21-year old woman traveler to Africa who suffered from irreversible loss of vision along with optic neuritis and encephalitis upon receiving vaccinations for yellow fever, hepatitis A, and B. The causal factor among the multiple vaccines could not be resolved during the investigation [11]. To our knowledge, there have been no further reports of eye infections, and therefore, the involvement of YFV in causing ocular complications is still not clear.

### 2.2. Japanese Encephalitis Virus (JEV)

JEV is the causal agent for Japanese Encephalitis, which can lead to severe neurological damage [47] and is known to cause 30,000 to 50,000 cases each year in the Pacific and Asia. Symptoms depend on the affected part of the nervous system and include early symptoms, such as non-specific febrile illness, diarrhea and rigor, followed by reduced levels of consciousness, seizures, headache, photophobia, and vomiting [13]. Late symptoms could include poliomyelitis-like flaccid paralysis [12] and parkinsonian syndrome. Patients exhibit the classic description of Japanese encephalitis-dull, flat, mask-like face with wide, unblinking eyes, tremor, generalized hypertonia, cogwheel rigidity, and other complications in locomotion [13]. The frequency of seizures increase with the severity of the disease [14]. In fatal cases of JEV, pathological changes are polymorphic and diffuse, involving different parts of the nervous system where the brain shows a severe degree of congestion in vasculature, microglial proliferation, and gliomesenchymal nodules formation, cystic necrosis in focal or confluent areas, cerebral edema, and trans-compartmental shift which can also lead to acute coma [15,16].

There has been a drastic reduction in the occurrence of JEV induced encephalitis cases since the use of a live attenuated vaccine (LAV) for humans [48]. There has been only a single case study published on a 53-year old woman infected with JEV leading to blurred vision with retinal hemorrhage and a clinical presentation of ocular fundus [17]. In vitro studies on JEV have shown that virus infection leads to the production of a macrophage-derived neutrophil chemotactic factor, which alters the blood-retinal barrier and thereby might have been a cause for the observed retinal hemorrhage [17,18].

### 2.3. Kyasanur Forest Disease Virus (KFDV)

KFDV was first reported in 1957 from Kyasanur forest in Karnataka, India and is prevalent in South Asia. It is transmitted to humans and animals by the bite of infected ticks (*Haemophysalis spinigera*) [49]. It causes biphasic illness composed of acute and convalescent phase [50]. Chief pathological features include hemorrhagic pneumonitis, hepatomegaly and parenchymatic degeneration, nephrosis, characteristic reticuloendothelial cells in spleen and liver [19] along with leucopenia, thrombocytopenia, reduced red blood cells and elevated levels of liver enzymes [20]. The second non-viremic phase may include bradycardia, meningoencephalitis, hemorrhagic fever manifestations, conjunctival inflammation, coma and neurological complications such as mental disturbance, light-headedness, stiff neck, abnormality of reflexes, confusion, and tremors [19,21]. KFDV has been classified as a risk group 4 pathogens and NIAID (National Institute of Allergy and Infectious Diseases) Category C priority pathogen due to its extreme pathogenicity and lack of US FDA approved vaccines and drugs [21].

There are around 400–500 cases reported annually during the past five decades, and the ophthalmic presentation of KFDV includes hemorrhages in the conjunctiva, vitreous humor, and retina, mild iritis, the opacity of lens and keratitis [20,22,23,24]. KFDV has only been reported from India, and there have been no further studies to understand the ocular anomalies and the reason behind the complications. Currently, there are no experimental models to study its pathogenesis.

### 2.4. West Nile Virus (WNV)

Smithburn et al. first isolated WNV, a neuro-invasive flavivirus, from the West Nile district of Uganda in 1937 [51]. The disease has gained recent global attention due to its reported multiple outbreaks in Africa, Asia, Europe, and the United States. The incubation period for the infection in humans range from 3 to 14 days along with three different systemic disease presentations: asymptomatic, fever and meningoencephalitis. Severe, potentially lethal, involvement of neurological complications (encephalitis, meningoencephalitis, acute flaccid paralysis—poliomyelitis-like, Guillain–Barré syndrome and optic neuritis) had been limited to 1% in the past, but with time, WNV has increased its severity. WNV meningoencephalitis may be characterized by a headache, photophobia, back pain, confusion, and fever.

The ocular involvement following WNV infection was first reported during 2002–2003 with chorioretinitis, anterior uveitis, retinal vasculitis, optic neuritis, and congenital chorioretinal scarring [6]. Around 80% of the WNV infected patients with neurological complications suffered from multifocal chorioretinitis without any ocular symptoms or vaguely reduced vision. The multifocal pattern has been regarded as an early diagnostic marker for WNV infection with meningoencephalitis. Currently, there are no vaccines or antivirals against WNV infection. Clinical cases usually recover within a week and upon administration of steroids [52].

A prospective case study in India revealed that 70% of positive cases with WNV infection reported additional ocular complications apart from what was initially reported. The fundus examination revealed discrete superficial white retinitis, arteritis, phlebitis, and retinal hemorrhages with or without a macular star [5]. Moreover, areas of retinal inflammation with unclear borders, vascular and optic disc leakage, vessel wall staining, or capillary non-perfusion were also observed. One of the patients with diabetes exhibited choroidal inflammation.

Other various ocular complications reported in the past are iridocyclitis in the absence of chorioretinitis, retinitis, retinal hemorrhages, focal or diffuse vascular sheathing, vascular leakage, macular edema, occlusive vasculitis, and segmental wedge-shaped zones of atrophy and mottling of the retinal pigment epithelium [53,54,55,56]. WNV-associated optic nerve involvement may occur, including optic neuritis, neuroretinitis, optic disc swelling. WNV infection has been rarely associated with Opsoclonus-myoclonus syndrome (OMS), also known as the dancing eye syndrome in patients with rapid, involuntary, multifactorial, conjugated fast eye movements persisting during sleep [7,8]. The exact mechanisms related to optic neuropathy related to WNV are still unknown.

### 2.5. Dengue Virus (DENV)

DENV has been involved in causing epidemics throughout the tropics and subtropics since the 1950s, with over one-third of the world’s population living at risk of infection. The transmission of DENV occurs between humans and *Aedes* mosquitoes, with incubation periods between 3 to 10 days and symptoms lasting from 3 to 7 days [57,58,59]. There are four serotypes of DENV reported, DENV-1, DENV-2, DENV-3, and DENV-4 [60], that can cause dengue fever (DF) as well as severe forms of dengue hemorrhagic fever (DHF) and dengue shock syndrome (DSS).

The symptoms during dengue fever usually include high fever, severe headache, retro-orbital pain, arthralgia, myalgia, nausea, vomiting, and rash. A severe form of dengue may be lethal to individuals with symptoms including bleeding gums, restlessness, fatigue, blood in vomit, and thrombocytopenia [25]. Severe forms of DENV infection mostly occur due to antibody-dependent enhancement (ADE), where pre-existing antibodies from a primary infection bind to an infecting DENV particle during secondary infection with a different DENV serotype [61]. While only 5% develop severe, life-threating infections, most dengue infections are asymptomatic. Recently, DENV NS1 protein has also been shown to be responsible for causing vascular leakage in both in vitro and in vivo models and is being used as a marker to detect DENV infection in DHF patients [62,63,64]. The viral NS1 protein is secreted from the cells and stays in the blood circulation of patients even after the fever and viral nucleic acid subside. At the time when severe dengue hemorrhage begins, NS1 protein levels correlate well with the degree of thrombocytopenia [65].

Traditionally, ocular pathology in dengue fever was thought to be uncommon; however, its involvement of ocular complications is now being recognized increasingly as it leads to permanent visual impairment in certain cases. Clinical studies have not seen any disease correlation with age, sex or ethnicity as risk factors [26]. Factors which have been postulated in the pathogenesis of dengue ocular diseases include viral virulence, serotypes, mutations, host susceptibility and geographic factor. Dengue eye disease can be unilateral or bilateral and the onset of ocular symptoms range from 2 to 5 days after the onset of fever and most ocular symptoms have been noted within one day after the peak of thrombocytopenia [27]. One study reported that 10% of 160 DENV seropositive hospitalized patients had maculopathy [26]. The main ocular complaints are eye pain, retro-ocular pain, blurring of vision, diplopia, foreign body sensation, photopsia, floaters, and metamorphopsia. In addition to this, other ocular symptoms include blurred vision floaters, subconjunctival hemorrhage, uveitis, and vitritis etc. (Table 2) [26,27,28,29,30,31,32,33,34,35]. Significant predictors of ocular symptoms included leukopenia and hypoalbuminemia, which could predispose patients to an opportunistic infection of ocular tissues and hyper permeability [66].

Dengue maculopathy is well recognized and studied more than other ocular manifestations, and it has been seen to be serotype and geography-related. There have been very few studies to understand the mechanism behind dengue-induced ocular implications which limit our understanding of the disease pathology. There is only one study relating maculopathy to be serotype specific with DENV-1 epidemic causing 10% incidence while there were no cases during DENV-2 epidemic [67]. Macular edema and macular hemorrhage were common findings in symptomatic patients with maculopathy.

While cellular and molecular mechanisms to understand systemic dengue has been extensively studied, there has been no studies of DENV in vitro as well as in vivo to understand the disease pathology, risk factors of eye disease and preventive measures. The serious threat to vision is dengue retinopathy, including retinal vasculopathy and macular edema. The mechanism of retinopathy is not clearly understood, but observations in patients implicate the involvement of retinal pigment epithelium (RPE) and endothelial cells. Smith et al. 2017 have recently shown that retinal epithelial and endothelial cells are susceptible to DENV infection with cytopathic effects in epithelial cells. The infection decreased the epithelial barrier integrity while the endothelial junctions were intact which correlated with the clinically observed loss of RPE in DENV infected patients [68].

Research on DENV vaccine has been a challenging path because there are four known serotypes and the associated complications of partial immunity leading to increase disease severity due to ADE. Live attenuated vaccines based on recombinant attenuated DENV serotypes (NIH) or recombinant yellow fever virus (Sanofi) or DENV-2 (Takeda) constructs expressing prM and Envelope genes from four serotypes, are currently in phase 3 or 4 clinical trials. The leading candidate, Dengvaxia, developed by Sanofi Pasteur, has been approved for use in individuals above the age of 9 years in Brazil, Mexico and the Philippines [69,70,71].

### 2.6. Zika Virus (ZIKV)

Zika virus was first isolated from a rhesus macaque from the Zika forest in Uganda, 1947 [72], with its first human detection in 1952 [72,73]. ZIKV then spread throughout parts of Africa and Asia, and in 2007 a ZIKV epidemic on Yap Island in Micronesia had 49 confirmed cases [74]. Over the last 2 years, it has raised a global alarm as it infected people in more than 70 countries causing severe deformities in newborns and neurological diseases in adults [75]. Apart from being transmitted by the vector (mosquitoes), ZIKV is capable of vertical transmission in humans during pregnancy or delivery, through sexual contact, or through contaminated blood transfusions. The incubation period for ZIKV ranges from 3 to 14 days while the symptoms last from 3 to 7 days. The risk of transmission of infection is escalated because 80% of infected adults remain asymptomatic. The usually visible signs of infection are mild and include fever, rash, headache, joint pain, conjunctivitis, muscle pain, and may result in Guillain-Barre syndrome. Congenitally contracted ZIKV causes birth defects in newborns such as microcephaly, impaired brain development, hearing loss, seizures, impaired joint movement, facial deformities, and vision problems [76].

In the eyes of adults, ZIKV presents most commonly as non-purulent conjunctivitis; however, more serious findings, such as the disruption of the RPE and iridocyclitis, have been reported in healthy and immunocompromised patients [42,77,78]. The first report on Congenital Zika Syndrome (CZS) had originated from Brazil describing ophthalmologic findings in three children with microcephaly caused by presumed ZIKV infection. There has been a steep increase in reported cases in the pathology of ocular complication in CZS, thereafter. Children present with gross macular pigment mottling, foveal reflex loss, macular neuroretinal atrophy, and fundoscopic alterations in the macular regions [79]. There have also been reports of chorioretinal atrophy, optic neuritis, retinal hemorrhaging, retinal mottling, iris coloboma, lens subluxation, gross macular pigment mottling, optic nerve hyperplasia, macular chorioretinal atrophy in other reported CZS cases [36,37,38,39,40]. Retinal abnormalities were also reported without microcephaly indicating the capability of ZIKV to cause ocular complications by direct infection [37]. The first study to reveal the ocular histopathologic findings of ocular tissue samples from deceased CZS fetuses by Fernandez et al., 2017 revealed pupillary membranes, immature anterior chamber angles, loss of pigment and thinning of the retinal pigment epithelium, choroidal thinning, undifferentiated nuclear layers of the retina, and a perivascular inflammatory infiltrate within the choroid. The viral antigen could be detected in iris, neural retina, choroid and the optic nerve [80].

Currently, the long-term effects of these infections are unknown, but a report in an immunocompromised patient indicates that lesions can be persistent [77]. ZIKV has been isolated from conjunctival swabs of infected patients which indicates its ability to infect the peri-ocular tissues and transmit through ocular secretions [81]. To date, there are thirteen open clinical trials at different phases, testing various concepts of ZIKV ranging from DNA vaccines, mRNA vaccines, Purified Inactivated Virus (PIV) vaccines and viral-vector-based vaccines [82,83].

## 3. Experimental Models of Ocular ZIKV Complications

To alleviate the disease burden associated with ocular viral infections, it is necessary to focus today’s research on disease control and therapeutic strategies. An animal model for a disease would prove to be a valuable tool to shed light on the pathophysiology of infection and facilitate the assessment of therapeutics and vaccines in the offing.

### 3.1. In Vivo Models

There have been several animal models used to mimic the ZIKV infection. The wild-type C57BL/6, BALB/c, and CD-1 mice are resistant to flavivirus infections [84,85,86]. Most of the studies used immunocompromised mice, both type I and type II interferon knockout mice (AG129), for efficient infection and viral replication to mimic human symptoms [86,87,88,89,90,91]. The first model of ocular complication during ZIKV infection in mice was described by Miner et al., where subcutaneous injection of the virus in IFNAR^−/−^ mice caused panuveitis and shedding of viral RNA in tears, but there was complete absence of live virus in ocular tissues and retina, as seen in humans [88]. There were no histological abnormalities evident in the eyes of congenitally infected IFNAR^+/−^ fetuses from C57BL/6 IFNAR1^−/−^ dams [88]. Direct inoculation of the virus in the organ/tissue of interest has been another approach used by various groups [92,93]. Singh et al. have developed a new model of ocular ZIKV infection to mimic human disease conditions by injecting the ZIKV intravitreally in the eyes of immunocompetent C57BL/6 mice or ISG15 knockout mice [94]. This model could show the observed pathological findings of retinal pigment epithelium atrophy and pigment clumping/mottling in humans. Another successful mouse model showed ocular infections mimicking humans upon subcutaneous injection of ZIKV in 1 day old pups (p1) [75]. The model showed presence of virus in the ocular tissues till 30 days pi while the inflammation subsided by 60 dpi. Therefore, these models could be used to study ocular complications in ZIKV as well as other viral infections to understand the viral pathogenesis in the ocular tissue.

In mouse models, ZIKV has been shown to infect multiple ocular cell types and that ZIKV inoculated mice developed conjunctivitis, pan-uveitis, infection of the cornea, iris, optic nerve, retina, as well as the detection of viral RNA in tears [88,94]. These studies exhibit the ability of ZIKV to infect previously unexplored cell types of the eye, and the need for research investigating these relationships is of paramount importance. The studies indicate the spread of the virus via two modes: hematogenic or axonal which is also supported by Fernandez et al. [80]. The virus could reach the fetal circulation from the placenta and infect the RPE, retinal endothelial cells, and the retina [94]. The other possible pathway would be through axonal transport into the eye along the optic nerve [95,96], leading to ganglion cell layer (GCL) loss, foveal maldevelopment, and central chorioretinal abnormalities [97]. How ZIKV and other flaviviruses enter the eye in vivo remains to be determined.

### 3.2. In Vitro Models

Since wild-type mice are not susceptible to ZIKV, most in vitro studies have used primary human cells or established cell lines. For example, Singh et al. have recently reported that retinal cell types lining the BRB are susceptible to ZIKV infection and cause cell death by activating Caspase 3 [94]. Zhao et al. and Aleman et al. have independently shown the Muller glia cells to be the primary target of ZIKV infection leading to decreased neurotropic functions and increased pro-inflammatory cytokines post infection with the help of murine models [96,98]. ZIKV infection in RPE was shown to disrupt its cell to cell adhesion and barrier properties [99]. Similarly, ZIKV was found to infect human fetal retinal pigment epithelial cells (FRPE), iPSC-derived retinal stem cells (iRSCs), and retinal cup (RC) organoids [100]. The underlying mechanisms and detailed sketch of the breach of BRB to cause the ocular pathology has not been addressed till now. Currently, the field of ZIKV research has been undecided on the role of the protein tyrosine kinase TAM receptors—TYRO3, AXL, and MER—in ZIKV entry into cells. The TAM receptors have been shown to be upregulated in the retinal epithelial and endothelial primary cells during ZIKV infection but their roles are still under debate [94].

## 4. Interaction with Blood-Retinal Barrier (BRB)

The BRB forms as an extension of the blood-brain barrier (BBB), which aids in the separation of the internal environment of the eye from the systemic vascular system. The BRB has two modules: an inner barrier, formed by retinal endothelial cells, and an outer barrier, formed by the RPE layer along with Bruch’s membrane and choriocapillaries [94]. The outer BRB shields the neuroretina from blood-borne pathogens including viruses. Therefore, disruption of the RPE could create a route of entry for virus present in fenestrated choroidal capillaries to enter the tissues of the eye. The BRB is limited internally by tight junctions between the endothelial cells underlining the retinal capillaries whereas the outer barrier is formed of tight junctions between the cells of RPE, separating the choroid system from the sensory retina [101].

The development and maintenance of the BRB is needed for healthy vision and the loss of the BRB leads to the pathology of a variety of retinal diseases. A balance of the vascular endothelial and epithelial mechanisms of the BRB supports the specialized neural retina environment. The vascular endothelium and the pigment epithelium layer of the retina possess a highly developed complex of inter cellular junction complexes including the adherens and tight junctions [102,103]. These junctions impart a high degree of barrier permeability to the solute and fluid across the retina. An understanding of BRB regulation during viral infection will allow the development of therapies aimed at restoring the compromised barrier or manipulating the barrier for specific transport of therapies.

ZIKV is permissive to all cell types comprising the different layers of the eye except the photoreceptor cells and causes similar pathological findings as in human patients with chorioretinal atrophy, pigment mottling/clumping [94]. There are two models proposed for the infection and entry of ZIKV into the eye: (a) cell death caused by ZIKV infection in the outer BRB might form the portal of entry of the virus into the eye and infect the inner layers, thereby causing inflammation and vision loss in severe infected cases [94]; (b) ZIKV could possibly enter via the retinal arteries (inner BRB) which leads to infection of the retinal endothelial cells and pericytes and the virus enters the outer BRB via the choroid capillaries [104].

### 4.1. Modulation of Retinal Innate and Adaptive Immunity

RPE cells are multi-functional and help in transport of nutrients and waste from the retina across the choroid and impart adhesive properties to the retina (Bok, 1995, 1993). It forms the first line of defense against pathogens in the retina and has a role in innate as well as adaptive immunity [105]. These cells have been identified as an ideal target for infectious agents such as Cytomegalovirus, *Toxoplasma gondii*, Coronavirus, Zika virus [94,105]. They produce a variety of cytokines (IL6, IL8, MCP-1), chemokines, growth factors, and act as Antigen Presenting Cells (APC) in the retina upon a pathogen assault [106,107,108]. Few immune cells reside within the ocular tissues [41,109]. Upon infection, the peripheral immune cells can gain access to the eye leading to local inflammation [110]. Despite the upsurge of interest in the RPE cell and its critical role in retinal health and disease, the exact mechanisms of how the RPE cells participate in the regulation of the blood-retinal barrier remain largely unknown. The retina also contains specialized myeloid cells (microglia), similar to brain microglia and the central and peripheral rims of the retina contain a small population of DCIR^+^ MHC Class II^hi^ DCs, as does the corneal periphery [111]. In addition to neurons, retina contains glial cells (microglia and Muller glia) and astrocytes which not only provide structural support but play an important role in evoking retinal innate responses to injury or infectious stimuli. How these retinal residential glial cells regulate innate immunity is an active area of investigation in our laboratory. Similarly, cells lining the BRB, also contribute to retinal immune response to microbial infections.

ZIKV infection of RPE, Muller glia and the retinal endothelial cells leads to an increase in the markers of innate immune response along with interferon and interferon stimulated gene response in a time dependent manner and causes chorioretinal atrophy, RPE mottling and cell death in in vitro as well as in vivo models [75,94,112]. ZIKV induces the expression of AXL in all the retinal cell types except the photoreceptor cells and the TAM receptor seems to be the dominant receptor used for the infection of the retinal cell types. ZIKV infection leads to an increase in innate immune response with an increase in TLR3 expression along with an increase in the expression of other viral recognition receptors—MDA5, RIG-I. There is an increase in ocular pathology due to the inflammation caused by significant production of RANTES and an increase in the expression of inflammatory genes *TNFA, IL1B, CXCL10, CCL5* leading to ocular inflammation [94,104]. The infection also leads to elevated levels of granzyme B, perforin, IFN and IFN stimulated genes—*OAS2*, *ISG15*, and *MX1* [75,94].

Recently, Manangeeswaran et al., 2018 reported that subcutaneous infection can also lead to symptomatic posterior uveitis that could replicate the infection in the patients by employing wild-type B6 mouse model [75]. The model demonstrated the preferential infection of cornea and retina causing chorioretinal lesions upon ZIKV infection. The infection elicits an inflammatory response characterized by increased local chemokine expression, infiltration of neutrophils, APCs, natural killer cells and CD4 and CD8 cells in the later stage. The infection subsides by 30 dpi while the cytotoxic T cells remained in the eyes along with the expression of several chemokines. The study also showed that the cornea and retina had higher levels of chemokines associated with the infiltration of CD45+ cells along with biomarkers for APCs (CD86, B2m, H2-EB1) and T-cell infiltration (CD3, CD4, GITR, CD40L, Fas-L) causing increased cytotoxicity in the infected tissues. This new model of study could prove to be very helpful in dissecting the mechanism of the breach of BRB during ZIKV infection and the various alterations occurring in the ocular tissues as it mimics vector bite followed by infection and inflammation in the eye similar to symptoms in human.

During ZIKV infection, ISG15 acts as a key player in confining viral replication in the retina/eye mostly by positive loop regulation of interferon signaling. The backfire of the antiviral immunity in the infected host leads to a huge amount of tissue inflammation and tissue damage leading to vision loss and further complications which has also been evident during the fetus development [113]. Another parallel study on primary Muller glia cells demonstrated their high susceptibility to ZIKV infection and induce a robust inflammatory response. They activate several intracellular pathways, including ERK, p38MAPK, NF-kB, STAT3 and ER stress, thereby influencing the differential expression of growth and inflammatory factors. The p38MAPK has been shown to be strongly controlling the expression of inflammatory pathways and has been proven to be highly potent during many viral infections, including ZIKV [112].

### 4.2. Modulation of Cellular Metabolism 

As viruses are non-living entities and do not possess their individual metabolism, they alter host cellular metabolic pathways for their optimal replication. The flaviviruses are known to subvert cholesterol homeostasis using multiple mechanisms to transform lipid droplets into their replication complexes with host membranes. To identify unique molecular signature of ZIKV infection in RPE, Singh et al., performed meta-analysis of ZIKV infected RPE cells and other related flaviviruses, DENV, JEV, WNV [114]. This led to the identification of a 43 genes signature referred to as core signature genes which are dysregulated upon ZIKV infection and not by the other flaviviruses tested. The validation of some of the identified genes in the signature revealed that ZIKV modulated (upregulation) their expression at relatively higher levels than DENV. Interestingly, the pathway analysis revealed that ZIKV alters cellular metabolism involving SH3/SH2 adaptor activity, lipid metabolism and ceramide metabolism. The *SH2B3* gene may help in evading the immune system by attenuating the inflammatory response and the infiltration of innate immune cells to the site of infection [114]. The reduction in ALDH5A1 enzyme activity would lead to an increase in the endogenous levels of GHB (gamma-Hydroxybutyric acid) and GABA (gamma-aminobutyric acid) levels, leading to neurological manifestations of ZIKV infection such as microcephaly and Guillain-Barre Syndrome in adults.

ZIKV capsid protein has been recently shown to hijack host lipid metabolism for efficient viral replication and interact with nucleolar proteins to facilitate replication [115]. Among the highly altered genes, *ABCG1* and *ABCA1*, membrane transporters involved in cholesterol efflux and innate immune response, are among the top candidates. Moreover, inhibition of *ABCG1* resulted in reduced ZIKV replication in RPE cells [114]. As host cell lipids and cholesterol play an essential role in various stages of viral replication, including entry, uncoating, genome replication, assembly, and release, it is important to understand the molecules exploited by the virus and exploit the drug targets for therapeutic purposes. Recently studied global interactomics revealed the hijack of lipid metabolism machinery by the ZIKV Capsid protein and the interaction of NS2A and other ZIKV proteins with peroxisome-associated polypeptides that govern the lipid trafficking and innate immune regulation in host cells for the efficient replication of the virus [115]. The lipid metabolism machinery is centrally controlled by AMPK (5’ AMP activated protein kinase), one of the master regulators of various cellular metabolic pathways. AMPK has a prominent role during bacterial endophthalmitis with its activation by pharmacological drugs leading to a significant decrease in bacterial load in the infected eyes [116]. AMPK is known to be altered during flavivirus infection and has pro- as well as antiviral activity upon activation for different viruses [117]. Its role in ZIKV infection and ocular pathology has not been studied till now. Studies are ongoing in our laboratory to understand its role in viral replication and the breach of BRB by altering the intercellular junction machinery [118,119].

## 5. Conclusions

Ocular complications caused by flaviviruses and other viruses will have long-term economic, psychological and health implications. Therefore, a deeper understanding of the host–virus interaction and the viral pathogenesis in the eye will help in the discovery or re-purposing of therapeutic drugs to protect against viral-borne ocular abnormalities. As there are no known effective antiviral treatments or vaccines against emerging flaviviruses, infected patients can only be provided with palliative care upon diagnosis. The incidence of flavivirus infections can be reduced with supervised vector control and prevention of bites with vectors. A therapeutic approach to decrease viral load combined with a regulation of immune cascade response in the eye could save patients from detrimental ocular consequences in the future. Investigations in both immune-deficient and immune-competent mouse models of ZIKV infection may help to identify key host-pathogen factors and devise novel therapies to restrain the systemic and local inflammatory responses associated with ZIKV infection in the eye. The severity of ocular complications due to viral infections presents additional stress and challenges to communities that have already been devastated by the loss of life, community, and infrastructure. Flavivirus infections are seen increasingly in the endemic and non-endemic regions as the result of an increase in international travel, and therefore, ophthalmologists should have the requisite knowledge to diagnose and manage such patients and keep a track of the patient’s history for exposure to viral infections for early diagnosis of viral-related ocular complications. 

## 6. Future Directions

Viral infection epidemics pose significant challenges for healthcare and the world economy. Flavivirus infections causing ocular diseases will have significant long-term economic, psychological and health implications. Vision-related complications may be an underreported effect of flavivirus infection which has been a reason of global concern for many years. ZIKV infection and other related ocular complications in the recent epidemic in Brazil emphasizes the urgency to understand the virus pathogenesis in the eye and develop strategies to prevent potential vision loss due to viral infections. Viral infections may linger in immune privilege tissue/organs such as the central nervous system or the eye. The recent description of a patient recovering from Ebola, where the blood and urine tests were negative but ocular inflammation including anterior uveitis and vitritis continued, calls attention to the long-term effects of ocular infections and understand the role they might play in the replication cycle [120]. The precise pathogenesis of the ocular complications, development of specific antiviral therapy and vaccinations against these threatening flaviviruses are fields that require further research.

**Figure 3:**
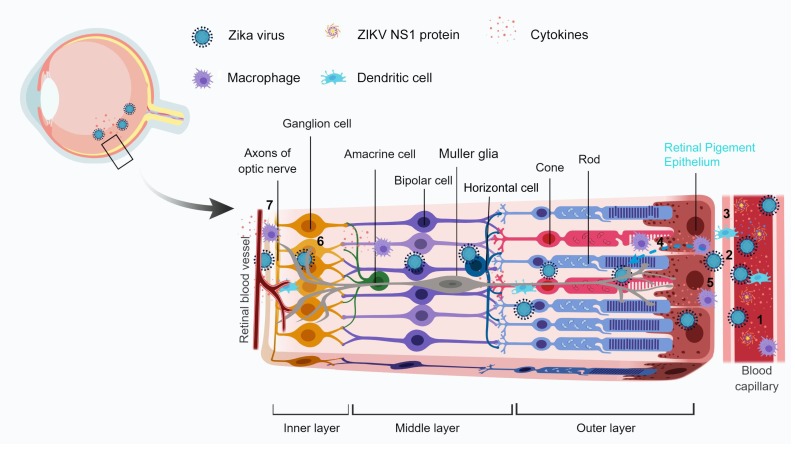
Probable mechanisms for the breach of blood-retinal barriers by ZIKV. Upon infection and peak viremia, there is an increased circulation of Zika virus, ZIKV NS1 protein, and immune cells in the blood (1). The virus in the retinal blood capillaries infect the endothelial lining (2) and the immune cells reach the site of infection/inflammation by diapedesis through the capillaries (3). It is followed by infection of RPE, the cell lining the outer BRB, resulting in chorioretinal atrophy. The viral infections might cause a BRB weakening by decreasing intercellular junction integrity. Being a neurotrophic virus, at later stages ZIKV can infect retinal Muller glia or neurons inside of the eye (4). The complications are worsened with the involvement of immune cells which get activated upon infection and release a “cytokine storm” as an antiviral response which damages the host cells by altering the barrier integrity (5). The virus along with the circulating immune cells can cross the inner BRB (retinal blood vessels) and infect neuronal cells such as ganglion cells (6, 7). The Image has been created with BioRender software.

There could be various possible theories to explain the factors involved in the breach of the blood-retinal barrier and the transmission of the Zika virus to the eye. In this section, we are trying to hypothesize some of the probable ones to understand the ZIKV disease model and the increase in the severity of the disease caused and their transmission route to the ocular tissues (Figure 3).

### 6.1. Possible Involvement of ADE

Since the transmission system of flaviviruses overlap, there is a high probability that many patients who have ZIKV infection have also had a previous exposure to at least one of the DENV serotypes. Likewise, many patients now exposed to ZIKV are highly likely to be exposed to at least one of the DENV serotypes in the future. There have been reports of an increase in the severity of ZIKV infection in individuals who have been infected with DENV in the recent past via a mechanism similar to ADE [121,122,123]. Therefore, pre-existing immunity against one flavivirus can affect clinical outcomes and diagnosis of the disease produced by infection with a heterologous flavivirus.

### 6.2. Possible Role of Secreted ZIKV NS1 Protein

Secreted DENV NS1 protein, a marker for DENV infection in patients, has been shown to be a causal factor for severe dengue and hemorrhage in cell culture and in vivo studies [63,64,124]. Zika virus, being a closely related member of the same family, may also share similar antigenic roles for secreted NS1 protein and may be a potential candidate in causing retinal hemorrhage and ocular complications by being a possible mediator in breaching the blood-retinal barrier.

### 6.3. Involvement of Immune Response and Cytokine Storm

The ocular complications are usually the outcome of a robust immune response from the host following an infection. During DENV infection, hemorrhage caused in tissues is mostly due to the cytokine storm from invading immune cells into tissues, thereby increasing severity of the disease [125,126,127]. The invading immune cells in the eye could also be exacerbating the severity of the complications and damaging the retina and blood vessels due to their response to control the infection. The inflammation and infiltrating cells may play a key role in clearing the virus but may also contribute to the development of lesions. Understanding the role of the immune response in the generation and persistence of the retinal lesions may enlighten the helpfulness of using immune suppressors, such as corticoids, during the later stages in the disease.

### 6.4. Involvement of the Altered Host Machinery

Flaviviruses employ the host lipid metabolism machinery for its replication which makes it a potent target for therapeutic purpose [2,128,129]. Flaviviruses have been shown to impair the intercellular junction integrity via different cellular intermediates and cause a breach in the barrier via tight junction and adherens junction protein alteration and degradation. Little research has been conducted to understand the pathogenesis of ZIKV and how it breaches the blood-retinal barrier to cause ocular complications. Our recent study using a transcriptomic approach on retinal epithelial cells, a major component of the outer blood-retinal barrier, indicates the involvement of cholesterol metabolism in viral replication [114]. Targeting host cellular metabolism could alleviate ocular complications due to ZIKV infection.

Therefore, a deeper understanding of the ocular pathogenesis of viral diseases and their interaction with the various cell types involved in the blood-retinal barrier can etch a path towards the invention or re-purposing of therapeutic drugs to prevent vision loss in infected patients. The ocular complications during viral infections have been under-reported mostly due to the late or ignored investigation by ophthalmologists. Ophthalmologists have a crucial role to help in decreasing ocular complications by being aware of changing symptoms during flavivirus infections and having a complete patient history of their previous exposure to viral infections to rule out secondary infection complications.

## Figures and Tables

**Table 1:** Comparison of the major ocular findings among the different flaviviruses.

Ocular Complication	ZIKV	DENV	JEV	WNV	YFV	KFDV
**Conjunctivitis/keratitis**	+	+	−	−	−	+
**Macular mottling**	+	+	−	−	−	−
**Chorioretinal atrophy**	+ focal pigmentary clumping	+	−	−	−	+
**Optic nerve abnormalities**	+	+	−	+	+	−
**Cataract**	Hypoplasia, cupping, pallor	−	−	−	−	−
**Microphthalmia**	+	−	−	−	−	−
**Iris coloboma**	+	−	−	−	−	−
**Uveitis**	+	+	−	+	−	+
**Chorioretinitis**	+	+	−	−	−	+
**Retinal hemorrhage**	+	+	+	−	−	+

ZIKV—Zika virus; DENV—Dengue virus; JEV—Japanese Encephalitis virus; WNV—West Nile virus; YFV—Yellow Fever virus; KDFV—Kyasanur Forest Disease virus.

**Table 2:** Flaviviruses known to cause ocular disease in humans and their symptoms.

Virus	General Symptoms	Ocular Disease in Humans	References
**West Nile Virus**	headache, photophobia, back pain, confusion, fever, encephalitis, meningoencephalitis, acute flaccid paralysis—poliomyelitis-like, Guillain–Barré syndrome	chorioretinitis, anterior uveitis, retinal vasculitis, optic neuritis, and congenital chorioretinal scarring	[5,6,7,8]
**Yellow Fever Virus**	fever, chills, malaise, headache, lower back pain, generalized myalgia, nausea, and dizziness, vomiting, epigastric pain, prostration, and dehydration, petechiae, ecchymoses, epistaxis (bleeding of the gums), and the characteristic “black vomit” (gastrointestinal bleeding)	loss of vision, optic neuritis	[9,10,11]
**Japanese Encephalitis Virus**	Fever, headache, vomiting, fits, encephalitis, and coma	Blurred vision, retinal hemorrhage, ocular fundus	[12,13,14,15,16,17,18]
**Kyasanur Forest Disease Virus**	Frontal headache, fever, hemorrhagic pneumonitis, hepatomegaly and parenchymatic degeneration, nephrosis, characteristic reticulo-endothelial cells in spleen and liver along with leucopenia, thrombocytopenia, reduced red blood cells, bradycardia, meningoencephalitis, hemorrhagic fever manifestations, coma, mental disturbance, giddiness, stiff neck, abnormality of reflexes	hemorrhages in the conjunctiva, vitreous humor, and retina, mild iritis, the opacity of lens and keratitis	[19,20,21,22,23,24]
**Dengue Virus**	Fever, retro-orbital pain, myalgia, thrombocytopenia, severe abdominal pain, persistent vomiting, bleeding gums, restlessness	maculopathy, blurred vision, scotoma, floaters, subconjunctival hemorrhage, uveitis, vitritis, retinal hemorrhaging, retinal venular widening, higher retinal vascular dimension, retinal vascular sheathing, RPE mottling, tortuous vessels, acute macular neuroretinopathy, intraretinal macular, retinal edema, cotton wool spots, Roth’s spot, retinal detachment, retinochoroiditis, neuroretinitis, choroidal effusions, choroidal neovascularization, optic disc swelling and optic disc neuropathy, oculomotor nerve palsy, and panophthalmitis	[25,26,27,28,29,30,31,32,33,34,35]
**Zika Virus**	fever, rash, headache, joint pain, conjunctivitis, muscle pain, and may result in Guillain-Barre syndrome, microcephaly, hearing loss, seizures, impaired joint movement, facial deformities	gross macular pigment mottling, foveal reflex loss, and macular neuroretinal atrophy, chorioretinal atrophy, optic neuritis, retinal hemorrhaging, retinal mottling, iris coloboma, lens subluxation, gross macular pigment mottling, optic nerve hyperplasia, macular chorioretinal atrophy, anterior uveitis, and non-purulent conjunctivitis	[36,37,38,39,40,41,42,43]

## Glossary

Chorioretinitis	Inflammation of the choroid and retina of the eye. It is a form of posterior uveitis
Keratitis	Inflammation of the cornea
Iritis	Inflammation of the iris
Chorioretinal atrophy	A condition of the eye where both the choroid and retina are damaged. This causes them to wither away and stop working
Maculopathy	Any pathological condition of the macula, an area at the center of the retina that is associated with highly sensitive, accurate vision
Conjunctivitis	Inflammation of the conjunctiva of the eye
Uveitis	Inflammation of the uvea
Vitritis	Inflammation of the vitreous body
Optic neuritis	Inflammation that damages the optic nerve, a bundle of nerve fibers that transmits visual information from your eye to the brain
RPE	Retinal pigment epithelium cells
ADE	Antibody-Dependent enhancement
Scotoma	A partial loss of vision or a blind spot in an otherwise normal visual field
cotton wool spots	Fluffy white patches on the retina. They are caused by damage to nerve fibers and are a result of accumulations of axoplasmic material within the nerve fiber layer
Roth’s spot	Retinal hemorrhages with white or pale centers
Panophthalmitis	Inflammation of all coats of the eye including intraocular structures
iris coloboma	A hole in the iris
BRB	Blood-retinal barrier
