# Erratum: Singh, S. et al. Ocular Manifestations of Emerging Flaviviruses and the Blood–Retinal Barrier. *Viruses* 2018, *10*, 530

**DOI:** 10.3390/v11050475

**Published:** 2019-05-24

**Authors:** Sneha Singh, Dustin Farr, Ashok Kumar

**Affiliations:** 1 Department of Ophthalmology, Visual and Anatomical Sciences, Wayne State University, Detroit, MI 48201, USA; gq8860@wayne.edu; 2 Department of Biochemistry, Microbiology, and Immunology, Wayne State University, Detroit, MI 48201, USA; dfarr@med.wayne.edu

The authors wish to correct the following erratum in this paper [1].

The second author’s name was inadvertently excluded from the article. The corrected author and affiliation lines should be:

Sneha Singh ^1^, Dustin Farr ^2^, Ashok Kumar ^1,2,^*

^1^  Department of Ophthalmology, Visual and Anatomical Sciences, Wayne State University, Detroit, MI 48201, USA; gq8860@wayne.edu
^2^  Department of Biochemistry, Microbiology, and Immunology, Wayne State University, Detroit, MI 48201, USA; dfarr@med.wayne.edu

The change of authorship was caused by lack of communication between the listed authors and Dustin Farr. The corresponding author was not aware of Dustin Farr’s minor writing contributions to this review article until he was contacted by this author in March this year and reviewed the previous draft.

The authors apologize for any inconvenience this may have caused.

The change does not affect the scientific results. The manuscript will be updated, and the original will remain online on the article webpage, with a reference to this Erratum.

## References

[1] Singh S., Kumar A. (2018). Ocular Manifestations of Emerging Flaviviruses and the Blood-Retinal Barrier. Viruses.

